# Human-caused wolf mortality persists for years after discontinuation of hunting

**DOI:** 10.1038/s41598-023-38148-z

**Published:** 2023-07-08

**Authors:** Roman Teo Oliynyk

**Affiliations:** 1 Department of Genetics, Harvard Medical School, Boston, 02115 USA; 2 Department of Computer Science, University of Auckland, Auckland, 1010 New Zealand

**Keywords:** Biodiversity, Conservation biology, Ecosystem ecology, Population dynamics, Restoration ecology, Computational models, Data integration, Software, Statistical methods, Ecology, Zoology, Ecology

## Abstract

By the mid-twentieth century, wolves were nearly extinct in the lower 48 states, with a small number surviving in northern Minnesota. After wolves were placed on the endangered species list in 1973, the northern Minnesota wolf population increased and stabilized by the early 2000s. A wolf trophy hunt was introduced in 2012–2014 and then halted by a court order in December 2014. The Minnesota Department of Natural Resources collected wolf radiotelemetry data for the years 2004–2019. Statistical analysis showed that wolf mortality remained close to constant from 2004 until the initiation of the hunt, and that mortality doubled with the initiation of the first hunting and trapping season in 2012, remaining at a nearly constant elevated level through 2019. Notably, average annual wolf mortality increased from 21.7% before wolf hunting seasons (10.0% by human causes and 11.7% natural causes) to 43.4% (35.8% by human causes and 7.6% natural causes). The fine-grained statistical trend implies that human-caused mortality increased sharply during the hunting seasons, while natural mortality initially dropped. After the hunt’s discontinuation, human-caused mortality remained higher than prior to the hunting seasons throughout the five years of the available after-hunt radiotelemetry data.

## Introduction

By the mid-twentieth century, wolves were nearly extinct in the lower 48 states of the United States, with only 450–700 wolves remaining in northern Minnesota in the 1950s^1,2^. After wolves were protected by the federal Endangered Species Act of 1973, the northern Minnesota wolf population gradually increased and stabilized in the early 2000s^3^. In late December 2011, the US Fish and Wildlife Service removed wolves in the western Great Lakes region from the federal endangered species list^4^. The following year, Minnesota and other states established a wolf trophy hunting and trapping season, which was extended in 2013 and 2014^5–8^. In December 2014, a federal judge placed wolves back on the endangered species list, thereby halting further hunting and trapping in Minnesota^9^.

According to wolf population surveys conducted by the Minnesota Department of Natural Resources (MN DNR)^3,10–13^, wolf population estimates for the years 2012–2014 varied from approximately 2,200 to 2,400. For the entire 2004–2019 period, the estimated wolf population ranged from 3,020 in the 2003-04 winter count (the highest number of the period), to the low of 2,211 in first post-hunt winter count in 2012–13, and ending with estimated 2,596 wolves in 2019–20 winter count^3^. MN DNR *Minnesota Wolf Season Reports*^6–8^ accounted for 413 wolves killed in 2012, 238 in 2013, and 264 in 2014. Furthermore, the MN DNR collected radiotelemetry data on wolf movements and mortality between 2004 and 2019; see Chakrabarti et al.^14^ for a description of the telemetry data collection methods. In this paper, “hunting and trapping season” will often be abbreviated to “hunting season”—inclusive of trapping—for conciseness.

This study aimed to compare wolf survival and mortality for two specific time ranges: before and after the initiation of the first wolf hunting season in 2012. Such analysis may add to understanding of the trends associated with the introduction of wolf hunting and trapping seasons, and help improve the stewardship of wolf populations in Minnesota and globally. Statistical analysis showed that wolf mortality was close to constant from 2004 until the first hunting season, followed by a doubling of wolf mortality after the initiation of the first hunting season in 2012, with mortality remaining at a nearly constant elevated level through 2019. The finer-grained trend implies that human-caused mortality increased sharply during hunting seasons, while naturally attributable mortality initially dropped. After the discontinuation of hunting, human-caused wolf mortality persisted at a higher level than before the initiation of hunting.

## Results

A regression analysis was applied to MN DNR wolf radiotelemetry data for the 2004–2019 period, with number of radio-days for all wolves combined over 12-month periods, as described in the Methods (see Supplementary Table 1 for a summary of wolf radiotelemetry data by calendar year). The goal was to compare two time periods represented in the data. For brevity, the first period is called “before hunt,” which started in the middle of 2004 when the first two wolves were radio-collared for the MN DNR tracking study, with a larger, more representative number of radio-collared wolves being added between 2005 and October 31st, 2012—before the initiation of the first wolf hunting and trapping season. The second period, which is called “during-and-after hunt”, started on November 1st, 2012. The hunting season started in the first week of November, while late hunting and trapping continued in January 2013. Similarly, the second and third annual hunting seasons started in November of each respective year. Thus, statistical comparison years covered from November 1st in one year to October 31st in the next year (see Supplementary Table 2 for wolf radiotelemetry summary with time reference offset to November 1st). The regression discontinuity analysis, described below, validates the need for this choice of time frames.

**Table 1:** Wolf survival and mortality summary.

Period	Survival	Yearly mortality			R-days	Wolf deaths by cause	
		All	Human	Natural		Human	Natural
*All wolves*							
**Before**	0.783 (0.69–0.89)	21.7%	10.0%	11.7%	22373	6.92	8.08
**During-and-after**	0.566 (0.48–0.67)	43.4%	35.8%	7.6%	28233	36.30	7.70
All	0.653 (0.59–0.73)	34.7%	25.4%	9.3%	50606	43.22	15.78
*Adult*							
**Before**	0.773 (0.67–0.89)	22.7%	11.3%	11.3%	18440	6.50	6.50
**During-and-after**	0.574 (0.48–0.69)	42.6%	33.0%	9.6%	22341	26.32	7.68
All	0.656 (0.58–0.74)	34.4%	24.0%	10.4%	40781	32.82	14.18
*Male*							
**Before**	0.754 (0.61–0.93)	24.6%	14.1%	10.6%	9035	4.00	3.00
**During-and-after**	0.583 (0.45–0.76)	41.7%	23.8%	17.9%	10841	9.14	6.86
All	0.655 (0.55–0.78)	34.5%	19.7%	14.8%	19876	13.14	9.86
*Female*							
**Before**	0.792 (0.66–0.95)	20.8%	8.3%	12.5%	9405	2.40	3.60
**During-and-after**	0.565 (0.43–0.74)	43.5%	41.0%	2.6%	11500	16.94	1.06
All	0.658 (0.56–0.78)	34.2%	27.6%	6.6%	20905	19.34	4.66
*Juvenile*							
**Before**	0.831 (0.64–1.07)	16.9%	0.0%	16.9%	3933	0.00	2.00
**During-and-after**	0.538 (0.37–0.79)	46.2%	46.2%	0.0%	5892	10.00	0.00
All	0.640 (0.50–0.82)	36.0%	30.0%	6.0%	9825	10.00	2.00

Wolf hunting seasons always commenced in November of 2012–2014; therefore, all statistics are offset to start on November 1st of each year. The “before” period includes 2004 to October 31, 2012, while the “during-and-after” period includes November 1st, 2012 to the end of 2019. All years were calculated for the entire data period, combining before and during-and-after periods. The range in parentheses following “Survival” is the 95% confidence interval range. R-days are the number of radiotelemetry days for each time interval. Fractional wolf death counts are the result of six wolf deaths that were of unknown causes, imputed proportionately to the known mortality causes for each period.

Overall, 150 wolves were tracked for a combined total of more than 50,000 radio-days over a period of nearly 16 years. This amount of data resulted in accurate summary survival statistics for the entire period, and separately for before and during-and-after hunting periods (see Table 1).

**Figure 1:**
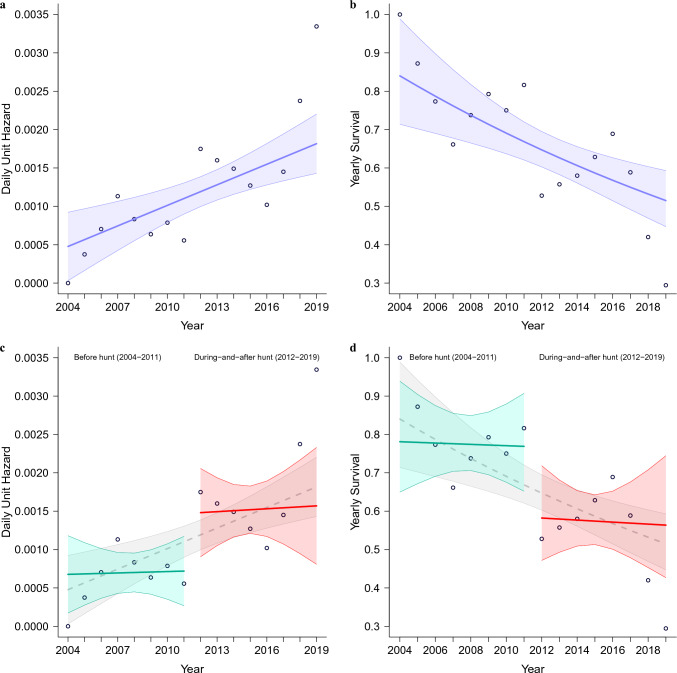
Survival statistics summary. Wolf hunting seasons always commenced in November 2012–2014; therefore, all statistics are offset to start on November 1st of each year. (**a**) Linear regression of daily unit hazard across the entire 2004–2019 period. Blue line—regression; blue shaded area—95% confidence interval. (**b**) Linear regression of yearly survival across the entire 2004–2019 period. Blue line—regression; blue shaded area—95% confidence interval. (**c**) Daily unit hazard regressions, separately for before (2004–2011) and during-and-after the first hunting season (2012–2019). Magenta line—trend before hunt, red line—trend during-and-after hunt; corresponding shaded areas—95% confidence interval. (**d**) Yearly survival regressions, separately for before (2004–2011) and during-and-after the first hunting season (2012–2019). Magenta line—trend before hunt, red line—trend during-and-after hunt; corresponding shaded areas—95% confidence interval. Circles represent consolidated unweighted yearly data points of hazard and survival respectively.

As shown in the section titled *All wolves* in Table 1, overall wolf mortality (all wolf ages and sexes) doubled from the before-hunt to during-and-after-hunt periods. The trends were similar for the *Adult*, *Male*, and *Female* sections, with male wolves showing slightly less than double the mortality increase from the before to during-and-after hunt periods, while overall female wolf mortality more than doubled and juvenile wolf mortality nearly tripled. Human-caused mortality increased for all wolf categories during and after the first hunting season; however, this increase was most notable for juvenile wolves, followed by female wolves. The proportion of natural cause mortality decreased during and after the hunt for all categories, with the exception of male wolves, whose natural mortality also increased—potentially due to conflicts caused by wolf pack disruption during and after hunts^15^.

Table 1 shows that all-cause mortality across wolf categories was within a close range among demographic groups, with juveniles showing the lowest mortality before the hunt and the highest mortality during-and-after the hunt, with all recorded during-and-after-hunt mortality being of human causes. Over the entire 16-year span of this study, 23 deaths were recorded for male wolves, 24 for female wolves, and 12 for juveniles. The 95% confidence intervals show the tightest fit for “All wolves” in Table 1. The 95% confidence intervals widened (along with fractionalizing radio-days) when splitting statistics by males, females, and juveniles, therefore discouraging the granular age-based regression on an annual scale separately for these categories. Thus, it made sense to perform the regression analysis on the overall tracked population.

A summary of the linear regression analysis is presented in Fig. 1. The daily unit hazard was calculated for each tracked year, linear regression was performed across the entire period (Fig. 1a), and corresponding yearly survival was derived from this result (Fig. 1b). The survival would be 0.83 in 2004, decreasing to 0.51 in 2019; however, there appears to be a distinct grouping of the survival data points (and daily unit hazard) for the periods before and during-and-after the hunt. Regression discontinuity analysis showed a significant trend cutoff point^16^ with a p-value of 0.02–0.04 exclusively within the October–December 2012 period (see in the Methods and Supplementary Note 3, particularly Supplementary Table 9). Independently, the statistical year offset for each 12-month period in Supplementary Note 2 showed the lowest standard error of linear regression for the statistical year starting on November 1st for all three regression periods: before hunt, during-and-after hunt, and even for a single regression over the entire period.

Trends tend to behave differently on both sides of a discontinuity, which called for separate regression analyses for these two periods. The regression analyses in Fig. 1c demonstrated relatively constant hazard/mortality periods before the hunt (shown in magenta), with daily hazard approximately doubling in the first hunt year and continuing on this new and almost constant hazard level during-and-after the hunt (red). A gray outline is retained for comparison with the entire 2004–2019 period regression in Fig. 1a. The corresponding analysis of yearly survival is presented in Fig. 1d, showing two roughly constant survival levels before and during-and-after the hunt, with a sharp 22% reduction in survival in 2012.

**Figure 2:**
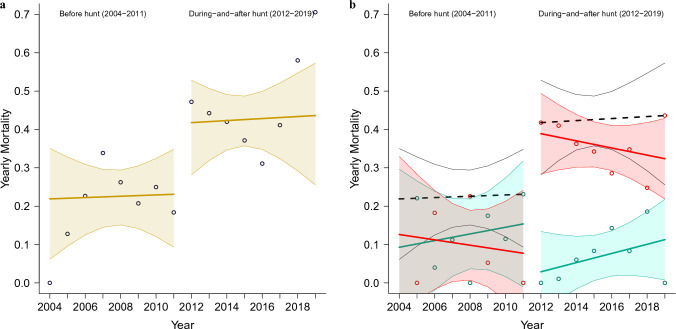
Wolf mortality for the periods before and during-and-after commencement of the first hunt. (**a**) All-cause mortality before and during-and-after hunting seasons. Beige line—trend before and during-and-after hunt indicated near the top of the figure; corresponding shaded areas—95% confidence interval. Circles represent consolidated unweighted yearly data points. (**b**) Same as (**a**), with mortality shared by natural and human causes. Magenta lines and data points—mortality trend by natural causes, red lines, and data points—mortality trend by human causes; corresponding shaded areas—95% confidence interval. Black dashed line—trend and confidence interval outlining the sum of all-cause mortality, as shown in plot (**a**). Before and during-and-after hunt periods are indicated near the top of the figure.

The mortality analysis presented in Fig. 2a matches the regression discontinuity analysis in Supplementary Fig 1. Fig. 2b presents the analysis with the data points and regression trend separated into human and natural causes, with their corresponding data points and confidence intervals, by cause of death using the overall mortality trend (dashed black line) as a reference. The human-caused and natural mortality numbers were low and close to equal before the hunt, both trending on an approximately constant level. The during-and-after-hunt period shows a distinct approximate tripling of human-caused mortality during the first hunt year, which only gradually decreased over the following years, with natural mortality notably dropping in the first hunt year and gradually increasing over the following years, altogether adding up to an approximate doubling of overall wolf mortality over the years following the initiation of hunting and trapping seasons. The additional regression discontinuity analysis over the during-and-after hunt period did not find a secondary trend cutoff, indicating the continuity of the during-and-after trend after the wolf hunts in Minnesota were stopped by the court order in December 2014 (see Supplementary Note 4).

## Discussion

In this research, linear regression analysis was applied to ascertain the patterns of wolf survival and mortality in Minnesota before and during-and-after the initiation of the 2012–2014 wolf hunting seasons based on wolf radiotelemetry statistics collected by the MN DNR during the 2004–2019 period. The regression discontinuity analysis, regression weighting fit when compared to the high-confidence extended period survival summaries, and the smallest residual standard errors of linear regression achieved when comparing 12-month periods starting on November 1st of each year (see Supplementary Note 2 and Supplementary Note 3) all point to statistically significant trend discontinuity timed with the initiation of the first hunting season in November 2012.

The period before wolf hunting seasons was characterized by near-constant wolf mortality averaging at 21.7% (see Table 1, with approximately equal proportions of mortality from human and natural causes (see Fig. 2b). After the initiation of the wolf hunting seasons in 2012, wolf mortality doubled to an average of 43.4% and remained close to constant for the during-and-after hunt period, with human-caused mortality becoming predominant, while the recorded natural mortality dropped during the wolf hunting seasons of 2012–2014. Human-caused mortality was highest during the hunting seasons, and while it started diminishing later, it remained higher than before the initiation of the hunting seasons throughout the year 2019. Notably, although overall mortality increased similarly for all wolf categories, human-caused mortality more than quadrupled for females and juveniles, and their natural mortality nearly vanished initially. For male wolves, while human-caused mortality less than doubled, the natural cause mortality also increased by 70%, which may have been caused by increased conflict and hunting difficulty due to pack disruption^15,17^ and pack disintegration^18^.

The present study’s findings of a rapid increase in wolf mortality following hunt initiation disagree with the only other recent paper using the same data set that was published by Chakrabarti et al.^14^, who “did not observe evidence that survival was markedly reduced during years when a regulated hunting and trapping season was implemented for wolves (years 2012–2014)”. Chakrabarti et al.^14^ applied Bayesian analysis in an attempt to determine which of the five smooth regression models tested would best fit the observed data (see Equations (2)–(6) in Chakrabarti et al.^14^). Smooth continuous models are not well suited for detecting trend discontinuities, and the model information criteria—DIC, WAIC, and LOOIC^19^—shown in Table 1^14^ differed only slightly among these five models, thereby indicating that no one model performed significantly better than any of the others. Chakrabarti et al.^14^ used statistical periods based on calendar years, which may have additionally been prone to masking discontinuous behavior. Compared to the Chakrabarti et al.^14^ paper, the novelties of the present work include:

1. The application of trend discontinuity analysis to test for trend cutoffs in periods before, during, and after the 2012–2014 wolf hunts (see Fig. 2). To the best of our knowledge, this is the first publication to use such a method to determine mortality trends over periods before, during, and after the introduction of wolf hunts.

2. The successful location of significant trend discontinuity (see Supplementary Note 3), co-timed with the start of the first wolf hunting season in November 2012, with two 8-year-long trends exhibiting nearly constant yearly mortality—before the hunts and during-and-after the hunts—where the yearly wolf mortality during-and-after the hunts was double that of before the hunts (see Fig. 2a and Table 1).

3. The finding that not only yearly wolf mortality doubled during the years with hunting seasons compared to the mortality levels before the hunts, but it remained constantly elevated for at least five years after the hunt discontinuation. While also validating the absence of statistically significant dip in wolf mortality during the years following the termination of wolf hunts in 2014.

4. The discovery that yearly human-caused wolf mortality more than tripled during the years with wolf hunts and remained elevated throughout the entire period during-and-after the hunts, even after the hunts were stopped by a court order in December 2014. At the same time, natural wolf mortality decreased during and after the hunts (see Fig. 2b and Table 1). The Chakrabarti et al.^14^ paper failed to report any of the above.

Human-caused mortality can be due to factors such as hunting, trapping, problem wolf elimination, illegal killing, poaching, and roadkill^14,20,21^. Chakrabarti et al.^14^ considered the increase in traffic death as one of the possible explanations for the observed increase in wolf mortality. However, it seems unlikely that the increase in traffic accidents could be a plausible explanation for the discontinuous trend switch observed in this study. Similarly, wolf dispersal events compel young wolves to navigate around or across risky, unfamiliar territories^14,22^. However, the discontinuous trend switch that showed doubling of mortality, timed with the commencement of the wolf hunting seasons, is unlikely to be explained by wolves gradually expanding into areas with denser human populations. Also, the locations of tracked wolf packs have not materially changed between the years 2007 and 2017 (as reported by MN DNR^11–13^), and wolves were recruited from established wolf territories rather than from newly expanded territories throughout the 2004–2019 period^3,10–13^.

Recent studies have documented human attitudes becoming more negative toward wolves after their killing was legalized for a period of time^23–26^. Remarkably, Fuller^27^ reported similarly high levels of wolf mortality in a 1980–1985 radio-tracking study of 81 wolves, with even higher human-caused mortality than that observed following the 2012–2014 hunting and trapping seasons—even though the wolf population was smaller in the 1980s. Fuller reported that during times when with “complete federal protection in 1974, controversy over wolves in Minnesota has abounded”^27^. A recent study in Wisconsin and Michigan concluded^23^ that liberalizing culling or hunting is more likely to increase illegal killing and poaching than reduce it, with controversy abounding again in Minnesota and neighboring states^28–31^.

In conclusion, before the initiation of the wolf hunting and trapping seasons of 2012–2014, wolf mortality was stable, with yearly wolf mortality being approximately 21.7% and caused by approximately equal proportions by natural and human causes, with natural causes being slightly more common. Something resembling a phase shift occurred with the initiation of the first hunting season, when wolf mortality doubled to 43.4%, became predominantly linked to human causes, and remained on such an elevated level over the 5 years following hunt discontinuation. On average, for the period during-and-after the initiation of wolf hunting seasons, human causes were linked to 35.8% of the entire wolf population each year, with natural mortality being responsible for 7.6% of the wolf population.

## Methods

### Description of the radiotelemetry data

MN DNR wolf telemetry data for 2004–2019 was received on request from the authors of Chakrabarti et al.^14^ (see Supplementary Data file *MnWolfSurvivalMortalityAge.csv*).

The survival analysis was performed by first aggregating the radio-days for each tracked wolf, with the cause of death date or censoring date parsed and sorted with the help of the C program *WolfDNR.exe*, which was written for this purpose by the author. Similarly to^14^, the pups and yearlings were counted together as juveniles for the final statistics since there were only 12 cases of juvenile mortality altogether. The juveniles’ radio-days were accounted for so that April 15th of each year was considered a “graduation day” for the older age category^14,27^. When crossing April 15th of a year at 2 years of age, these wolves’ radio-days were counted as adult radio-days thereafter. Notably, there was one occasion in the source file when pup W05-2270 was registered on May 6, 2005, at which age it was unlikely to be radio-collared. Thus, it was most likely a yearling, and an adjustment to the program was made to account for such scenarios, although it was the only exceptional case. Therefore, pup W05-2270 was reclassified as a yearling; within less than a year, it was censored while still yearling. The radiotelemetry data summaries parsed and arranged by the C program *WolfDNR.exe* (described further in this section) are visually explanatory, unlike the raw input CSV file. See the summary of a standard calendar year view in Supplementary Table 1 and statistical year offset to November 1st of each year in Supplementary Table 2, with corresponding radio-days and mortality counts.

### Imputation of wolf deaths from unknown causes

Of the 59 wolves reported dead in the dataset, the causes of mortality for 6 wolves were not determined. A preliminary analysis showed that mortalities within periods before the hunt and during-and-after the hunt remained at a relatively constant level for each of these periods. The ratio of human and natural mortalities was determined for before and during-and-after the hunt periods, and the missing mortality^32^ cause was imputed by assigning each of these mortalities’ fractional values for human and natural causes. This could be the case, for example, when necropsies were performed on carcasses in advanced stages of decomposition, making cause of death undetermined yet most likely to be attributed to the most common causes of death^33^. See the comparisons between Table 1 and corresponding Supplementary Table 11, where unknown deaths were omitted. The patterns appear to be qualitatively the same between the two representations, with  10% lower values across all numbers where the unknown deaths were omitted, which demonstrates the benefit of this imputation (see Supplementary Note 5).

### Survival analysis

The cumulative yearly wolf radio-days were calculated in *WolfDNR.exe* using the equation:

1 $$\begin{aligned} D(y) = \sum _{wolf\;Id=0}^{wolf\;Id\;max}\sum _{d=1}^{365} Wolf\;Day\;Array\;[wolf\;Id][365 * y + d], \end{aligned}$$

thus summing all entries for each individual wolf ID in the MN DNR data file that were accumulated above in *WolfDayArray* for the continuous count of days *d* of each year starting from 2004 (denoted as *y*), resulting in the sum of *D* for each year. The deaths or censored individuals, with their causes, wolf sexes, and ages, were added for each year in this same loop.

The average daily statistics were calculated using the maximum likelihood estimator (MLE)^34,35^. The periods were considered tracked daily (see also^14^), and wolf mortality considered established as the end date in the MN DNR data (see further treatment in discussion of regression discontinuity in Supplementary Note 3). The resulting survival numbers for this simple case scenario matched the initial value of Trent & Rongstad’s^36^ daily survival equation in the first MLE iteration:

2 $$\begin{aligned} S_d = (D - K) / D, \end{aligned}$$

where *D* represents the sum of radio-days for the given period and *K* represents recorded mortality counts during this period.

The yearly survival was counted for standard 365-day years:

3 $$\begin{aligned} S_y = S_d^{365}, \end{aligned}$$

where $S_y$ is yearly survival. Yearly mortality $M_y$ is complementary to yearly survival:

4 $$\begin{aligned} M_y = 1.0 - S_y. \end{aligned}$$

### Weighted linear regression analysis

There were 150 wolves tracked during the 2004–2019 data period with combined estimate of 50,606 radio-days, resulting in the average 337 radio-days per tracked wolf. As the radio-day coverage was not even over the years, additionally statistics recording zero wolf mortality in the statistical year 2004, weighted linear regression was required to prevent bias by less representative data^37^. Two approaches are possible in this data set: weighting by yearly variance or weighting by yearly radio days squared. Both methods resulted in a remarkably close outcome, as discussed in Supplementary Note 2 (see comparisons in Supplementary Table 3—Supplementary Table 8). The weighting by yearly radio-days squared almost precisely matched the higher confidence summary survival data in Table 1, and thus was chosen for this study. See Supplementary Note 2 for the evaluation and choice of linear regression weighting.

### Regression discontinuity analysis

The R script *WolfProc.R* iterated through each month of five consecutive years (2010–2014) and tested for the existence of regression discontinuity cutoff points^16,38,39^. The only sharp trend discontinuity cutoff period was found to span October, November, and December 2012, with significant p-values of 0.022, 0.039, and 0.041, respectively, when the mortality trend jumped to a  22% higher level than before and continued along this post-discontinuity trend line. Thus, the introduction of wolf hunting seasons in November 2012 corresponded with the statistically significant discontinuity in the wolf mortality trend (see in-depth treatment in Supplementary Note 3).

### Implementation of linear regression analysis

The linear regression with weighting was implemented using standard R libraries with the core regression model as follows:

5 $$\begin{aligned} wFit = lm(Y \sim X, data = df, weights = D^2), \end{aligned}$$

where *wFit* is the weighted fit, Y is the typical daily hazard values, *X* is the year range, *D* is the number of radio-days (measurements) per year, *K* is the recorded number of wolf deaths, and *df* is an R data frame containing input loaded from the CSV data file. A simple linear regression was then used to trend the fraction of mortality by natural and human causes.

### Code and executables

The data preparation and sorting were performed with help of the C program *WolfDNR.exe*, which outputs CSV files with radio-days, mortality counts, corresponding daily hazard (overall and by mortality cause), yearly survival, and mortality data points using the approach described above. The majority of the processing was performed using the R script *WolfProc.R*, which takes as an input the raw yearly summaries aggregated by *WolfDNR.exe*, performs linear regression and regression discontinuity analysis, extracts the relevant statistics, and outputs the figures and tables in PDF and LaTeX formats, as used in the manuscript. Two additional R scripts, *WolfSummaryTableBeforeAfter.R* and *WolfTableByYear.R*, output the remaining tables used in the manuscript. All of the above programs and code are available in the Supplementary Data, along with the batch files that allow to perform all the processing, starting with the MN DNR data set CSV file and then outputting the data tables and graphs used in this manuscript. Although this code is intended for use with the MN DNR data set, it can be easily adjusted for processing different datasets.

### Statistics and reproducibility

The survival analysis was performed on radiotelemetry data originally sourced from MN DNR and obtained on request from Chakrabarti et al.^14^. The linear regression fit and discontinuity analysis were performed using standard R libraries. The reported tables and figures include the 95% confidence intervals. The regression discontinuity analysis used a p-value significance of $\le 0.05$.

### Supplementary Information

Supplementary Information 1.

Supplementary Information 2.

## Data Availability

The input data, source code, executable, and batch files necessary to perform analysis are available in the Supplementary Data ZIP file.
