# Author Correction: Human-caused wolf mortality persists for years after discontinuation of hunting

**DOI:** 10.1038/s41598-023-40886-z

**Published:** 2023-08-24

**Authors:** Roman Teo Oliynyk

**Affiliations:** 1 Department of Genetics, Harvard Medical School, Boston, 02115 USA; 2 Department of Computer Science, University of Auckland, Auckland, 1010 New Zealand

Correction to:* Scientific Reports*, 10.1038/s41598-023-38148-z, published online 08 July 2023.

The Competing Interests section in the original version of this Article was incorrect.

“The author declares no competing interests.”

now reads:

“The author is board member of the advocacy group Howling For Wolves. The position did not influence the work on this article and the advocacy group was not involved in design, analysis, or interpretation of the research.”

The original Article has been corrected.

