# Supplementary material for: Human-caused wolf mortality persists for years after discontinuation of hunting
Source: Sci Rep. 2023 Jul 8;13:11084. doi: 10.1038/s41598-023-38148-z (PMC10329645; doi:10.1038/s41598-023-38148-z)
Supplement: Supplementary file 2 — Supplementary Information 2. [file 41598_2023_38148_MOESM2_ESM.pdf]

Human-caused wolf mortality persists for years after discontinuation of hunting *(Supplementary Information)*

Roman Teo Oliynyk[1,2*]

[1*]Department of Genetics, Harvard Medical School, Boston, MA, USA.

[2]Department of Computer Science, University of Auckland, Auckland, New Zealand.

Corresponding author(s). E-mail(s): roli573@aucklanduni.ac.nz;

Contents

Supplementary Note 1. MN DNR radiotelemetry data summary for the 2004–2019 period **2**

Supplementary Note 2. Fit of weighting methods used in linear regression **3**

Supplementary Note 3. Pinpointing the trend change timing via regression discontinuity analysis **8**

Supplementary Note 4. Absence of trend discontinuities following termination of the wolf hunting seasons **11**

Supplementary Note 5. Confirming the consistency of regression when wolf deaths by unknown causes are excluded **12**

Supplementary Note 1. MN DNR radiotelemetry data summary for the 2004–2019 period

Supplementary Table 1: Radiotelemetry summaries for standard calendar years starting **January 1st**.

		Deaths			Daily hazard	
**Year**	**R-days**	**all**	**human**	**natural**	**unit**	**variance**
2004	469	0	0.000	0.000	0e+00	0e+00
2005	1668	0	0.000	0.000	0e+00	0e+00
2006	2946	2	1.000	1.000	6.79e-04	2.3e-07
2007	4645	4	3.545	0.455	8.61e-04	1.85e-07
2008	2716	3	1.000	2.000	1.1e-03	4.06e-07
2009	1839	0	0.000	0.000	0e+00	0e+00
2010	3030	2	0.545	1.455	6.6e-04	2.18e-07
2011	2175	2	1.000	1.000	9.2e-04	4.22e-07
2012	4412	5	3.000	2.000	1.13e-03	2.57e-07
2013	2889	6	5.786	0.214	2.08e-03	7.17e-07
2014	4617	6	6.000	0.000	1.3e-03	2.81e-07
2015	4424	5	4.000	1.000	1.13e-03	2.55e-07
2016	4487	8	5.786	2.214	1.78e-03	3.97e-07
2017	3316	2	2.000	0.000	6.03e-04	1.82e-07
2018	4716	9	6.571	2.429	1.91e-03	4.04e-07
2019	2388	5	3.000	2.000	2.09e-03	8.75e-07

The R-days column title stands for radiotelemetry days. Fractional wolf death counts are the result of six wolf deaths with unknown causes, imputed proportionately to the known causes of mortality for each period.

Supplementary Table 2: Radiotelemetry summaries with the first day of the year offset to **November 1st**, the month of wolf hunting season commencement.

		Deaths			Daily hazard	
**Year**	**R-days**	**all**	**human**	**natural**	**unit**	**variance**
2004	1424	0	0.000	0.000	0e+00	0e+00
2005	2672	1	0.000	1.000	3.74e-04	1.4e-07
2006	4262	3	2.462	0.538	7.04e-04	1.65e-07
2007	3533	4	2.000	2.000	1.13e-03	3.2e-07
2008	1200	1	1.000	0.000	8.33e-04	6.94e-07
2009	3143	2	0.462	1.538	6.36e-04	2.02e-07
2010	2541	2	1.000	1.000	7.87e-04	3.1e-07
2011	3598	2	0.000	2.000	5.56e-04	1.54e-07
2012	3431	6	6.000	0.000	1.75e-03	5.09e-07
2013	4375	7	6.825	0.175	1.6e-03	3.65e-07
2014	4694	7	6.000	1.000	1.49e-03	3.17e-07
2015	4720	6	4.825	1.175	1.27e-03	2.69e-07
2016	2941	3	2.000	1.000	1.02e-03	3.46e-07
2017	4825	7	5.650	1.350	1.45e-03	3e-07
2018	2948	7	4.000	3.000	2.37e-03	8.04e-07
2019	299	1	1.000	0.000	3.34e-03	1.11e-05

Wolf hunting seasons always commenced in November of 2012–2014; therefore, all statistics are offset to start each year on November 1st. This data arrangement was used as the basis for the regression analysis in this study. The R-days column title stands for radiotelemetry days. Fractional wolf death counts are the result of six wolf deaths with unknown causes, imputed proportionately to the known causes of mortality for each period.

Supplementary Note 2. Fit of weighting methods used in linear regression

The MN DNR radiotelemetry data were characterized by years with varying numbers of cumulative radio-days and wolf mortality events (as shown in Supplementary Table 1 and Supplementary Table 2). A common approach to limit bias from less representative data samples and outliers involves applying linear regression with weighting [1]. In statistical sampling, the inverse of variance is often used. However, in our data, years 2004, 2005, and 2009 in Supplementary Table 1 have 0 wolf deaths. Thus, no variance was calculated. Similarly, the year 2004 in Supplementary Table 2 has 0 wolf deaths (no variance) and quite a low number of radio-days, with one wolf death producing an outlier in the year 2019. Although the outliers were handled well by the variance weighting, the zero variance required an additional estimation of possible variance, perhaps from nearby valid entries. Additionally, with high-granularity radiotelemetry (as used in MN DNR data collection), the greater variance in years with higher wolf mortality may create its own bias. A second weighting approach involves using radio-days:

$$V = \frac{p(1-p)}{D} = \frac{\frac{K}{D}\frac{D-K}{D}}{D} \approx \frac{K}{D^2} \sim \frac{1}{D^2}, \tag{1}$$

where V is variance, p and $(1-p)$ are the probability fractions, D is the number of radio-days (measurements), and K is the count of wolf deaths in the radio-days period. With a large D and small K, $(D-K)/D \approx 1$, and $K/D^2 \sim 1/D^2$. The final step shows the use of the unbiased number of recorded deaths weighted by radio-days; thus, when weighting can be performed as $1/V$, it must be performed alternatively as D^2. The advantage of this second weighting approach is its consistency, and it does not require inventing a special treatment for the years with no recorded wolf mortality. The following comparisons show that this weighting method also allows a more precise match of the regression to the summary analysis in Table 1.

Finally, the R models for these two weights are implemented as follows:

$$wFit1 = lm(Y \sim X, data = df, weights = 1/V), \tag{2}$$

and

$$wFit2 = lm(Y \sim X, data = df, weights = D^2), \tag{3}$$

where wFit1 and wFit2 are the corresponding weighted fit scenarios for the linear regression, Y are the typical daily hazard values or survival, X is the year range, D is the number of radio-days (measurements) per year, and df is an R data frame with a loaded CSV input (see also Sheather [1]).

On the following three pages, the tables are grouped in comparison pairs for weighting using inverse variance and D^2 (see Supplementary Table 3–Supplementary Table 4, Supplementary Table 5–Supplementary Table 6, and Supplementary Table 7–Supplementary Table 8). For

completeness, these tables show the parameters of the linear regression with years offset to the first day of each month of the year. The most important columns are stdError (the standard error of the regression), r^2 (the goodness-of-fit), and mean survival, which is typically calculated as:

$$SurvM = (\prod_{t=Y_{min}}^{Y_{max}} S[t])^{1/T}, \tag{4}$$

where $SurvM$ is the mean survival over the subsequent number of years T in the regression interval Y_{min} to Y_{max}. As shown below, stdErr is comparatively lowest for years offset to start as of November 1st in all tables except Supplementary Table 4, where it is a close second. The regression parameter r^2 is a goodness-of-fit of the trend. In Supplementary Table 3–Supplementary Table 6 r^2 is low, representing a near-constant level of survival and mortality. For the entire dataset duration the best regression was also observed with a November 1st time frame offset.

The second equally important consideration for the weighting method is how well the mean survivals in these tables match the multiyear summaries in Table 1. Here, the summaries from sections in Table 1 compared to radio-days squared weighting in the regression analysis, are presented as follows (Supplementary Equation 3 scenario): for the 2004–2011 period, 0.783 vs 0.775; 2012–2019, 0.566 vs 0.573; all years' time intervals, 0.653 vs 0.658 — almost exact matches. Inverse variance weighting (Supplementary Equation 2 scenario) diverged further from the summary values in Table 1—showing 0.783 vs 0.804, 0.566 vs 0.582, and 0.653 vs 0.685, respectively—while exaggerating the summary survival in each case by a few percent.

Consequently, the regression analysis when using inverse variance weighting resulted in nearly identical but slightly underestimated mortality trend values. Thus, we used the weighting by radio-days squared in our reported analysis.

Supplementary Table 3: Linear regression fit parameters by assigned first month of the year for **years 2004–2011, inverse variance weighting**.

	Daily hazard fit				Survival		
**First month**	**stdError**	r^2	**Slope**	**Intercept**	**mean**	**begin**	**end**
Jan	0.000068	0.1207	0.000062	-0.1	0.796	0.861	0.735
Feb	0.000072	0.1469	0.000073	-0.1	0.799	0.877	0.727
Mar	0.000065	0.0418	-0.000034	0.07	0.802	0.768	0.837
Apr	0.000075	0.0130	-0.000021	0.04	0.792	0.771	0.814
May	0.000075	0.0083	-0.000017	0.03	0.795	0.778	0.812
Jun	0.000075	0.0067	-0.000015	0.03	0.799	0.784	0.814
Jul	0.000075	0.0058	-0.000014	0.03	0.804	0.790	0.818
Aug	0.000060	0.0030	0.000008	-0.02	0.802	0.811	0.794
Sep	0.000060	0.0162	-0.000019	0.04	0.796	0.777	0.816
Oct	0.000045	0.0031	-0.0000061	0.01	0.818	0.812	0.825
Nov	0.000042	0.1530	0.000043	-0.09	0.804	0.850	0.761
Dec	0.000047	0.0070	-0.0000097	0.02	0.792	0.782	0.802

Statistics for years: 2004 - 2011, linear regression weighted by inverse variance. 'stdError' is standard error, 'r^2', 'slope' and 'intercept' of the linear regression fit. Survival 'begin' and 'end' are survivals in the first and last year of the period.

Supplementary Table 4: Linear regression fit parameters by assigned first month of the year for **years 2004–2011, radio-days squared weighting**.

	Daily hazard fit				Survival		
**First month**	**stdError**	r^2	**Slope**	**Intercept**	**mean**	**begin**	**end**
Jan	0.000071	0.0182	0.000024	-0.05	0.764	0.788	0.742
Feb	0.000081	0.0261	0.000033	-0.06	0.772	0.804	0.740
Mar	0.000075	0.1014	-0.000062	0.1	0.756	0.698	0.818
Apr	0.000077	0.0538	-0.000045	0.09	0.754	0.712	0.799
May	0.000078	0.0392	-0.000039	0.08	0.757	0.720	0.795
Jun	0.000079	0.0430	-0.000041	0.08	0.760	0.721	0.801
Jul	0.000080	0.0525	-0.000046	0.09	0.764	0.721	0.811
Aug	0.000070	0.0061	-0.000013	0.03	0.760	0.747	0.773
Sep	0.000065	0.0344	-0.00003	0.06	0.766	0.737	0.797
Oct	0.000050	0.0495	-0.000028	0.06	0.779	0.752	0.807
Nov	0.000047	0.0027	0.0000061	-0.01	0.775	0.781	0.769
Dec	0.000043	0.0924	-0.000033	0.07	0.770	0.738	0.804

Statistics for years: 2004 - 2011, linear regression weighted by radio-days squared. 'stdError' is standard error, 'r^2', 'slope' and 'intercept' of the linear regression fit. Survival 'begin' and 'end' are survivals in the first and last year of the period.

Supplementary Table 5: Linear regression fit parameters by assigned first month of the year for **years 2012–2019, inverse variance weighting**.

	Daily hazard fit				Survival		
**First month**	**stdError**	r^2	**Slope**	**Intercept**	**mean**	**begin**	**end**
Jan	0.000096	0.0041	0.000015	-0.03	0.625	0.638	0.613
Feb	0.000113	0.0069	-0.000023	0.05	0.638	0.620	0.658
Mar	0.000139	0.0101	0.000034	-0.07	0.657	0.686	0.629
Apr	0.000104	0.0019	0.000011	-0.02	0.625	0.634	0.616
May	0.000109	0.0018	0.000011	-0.02	0.628	0.637	0.619
Jun	0.000113	0.0012	-0.0000098	0.02	0.626	0.618	0.634
Jul	0.000090	0.0005	-0.0000049	0.01	0.601	0.597	0.604
Aug	0.000088	0.0054	0.000016	-0.03	0.601	0.614	0.589
Sep	0.000092	0.0028	0.000012	-0.02	0.597	0.606	0.588
Oct	0.000087	0.0007	-0.0000058	0.01	0.587	0.583	0.592
Nov	0.000079	0.0003	0.0000034	-0.005	0.582	0.585	0.580
Dec	0.000119	0.0017	-0.000012	0.03	0.593	0.584	0.603

Statistics for years: 2012 - 2019, linear regression weighted by inverse variance. 'stdError' is standard error, 'r^2', 'slope' and 'intercept' of the linear regression fit. Survival 'begin' and 'end' are survivals in the first and last year of the period.

Supplementary Table 6: Linear regression fit parameters by assigned first month of the year for **years 2012–2019, radio-days squared weighting**.

	Daily hazard fit				Survival		
**First month**	**stdError**	r^2	**Slope**	**Intercept**	**mean**	**begin**	**end**
Jan	0.000079	0.1374	0.000077	-0.2	0.584	0.644	0.529
Feb	0.000098	0.0330	0.000044	-0.09	0.574	0.608	0.543
Mar	0.000107	0.0038	0.000016	-0.03	0.581	0.593	0.569
Apr	0.000097	0.0171	0.000031	-0.06	0.573	0.597	0.551
May	0.000106	0.0092	0.000025	-0.05	0.573	0.592	0.555
Jun	0.000106	0.0054	0.000019	-0.04	0.582	0.596	0.568
Jul	0.000088	0.0010	0.0000067	-0.01	0.582	0.587	0.577
Aug	0.000084	0.0092	0.00002	-0.04	0.587	0.602	0.573
Sep	0.000084	0.0003	0.0000038	-0.006	0.584	0.587	0.581
Oct	0.000080	0.0081	-0.000018	0.04	0.584	0.571	0.597
Nov	0.000069	0.0055	0.000013	-0.02	0.573	0.582	0.564
Dec	0.000117	0.0040	-0.000018	0.04	0.569	0.556	0.583

Statistics for years: 2012 - 2019, linear regression weighted by radio-days squared. 'stdError' is standard error, 'r^2', 'slope' and 'intercept' of the linear regression fit. Survival 'begin' and 'end' are survivals in the first and last year of the period.

Supplementary Table 7: Linear regression fit parameters by assigned first month of the year for **years 2004–2019, inverse variance weighting**

	Daily hazard fit				Survival		
**First month**	**stdError**	r^2	**Slope**	**Intercept**	**mean**	**begin**	**end**
Jan	0.000027	0.3511	0.000074	-0.1	0.704	0.862	0.575
Feb	0.000030	0.2679	0.000067	-0.1	0.712	0.856	0.593
Mar	0.000035	0.1838	0.000063	-0.1	0.737	0.874	0.621
Apr	0.000031	0.2591	0.000069	-0.1	0.707	0.853	0.586
May	0.000032	0.2480	0.000068	-0.1	0.708	0.854	0.587
Jun	0.000032	0.2432	0.000069	-0.1	0.709	0.855	0.587
Jul	0.000030	0.3481	0.000082	-0.2	0.698	0.873	0.557
Aug	0.000028	0.3981	0.000084	-0.2	0.701	0.883	0.556
Sep	0.000030	0.3567	0.000084	-0.2	0.702	0.884	0.558
Oct	0.000027	0.4305	0.000087	-0.2	0.705	0.896	0.555
Nov	0.000021	0.5959	0.000095	-0.2	0.685	0.889	0.528
Dec	0.000030	0.3397	0.000079	-0.2	0.693	0.861	0.558

Statistics for years: 2004 - 2019, linear regression weighted by inverse variance. 'stdError' is standard error, 'r^2', 'slope' and 'intercept' of the linear regression fit. Survival 'begin' and 'end' are survivals in the first and last year of the period. .

Supplementary Table 8: Linear regression fit parameters by assigned first month of the year for **years 2012–2019, radio-days squared weighting**.

	Daily hazard fit				Survival		
**First month**	**stdError**	r^2	**Slope**	**Intercept**	**mean**	**begin**	**end**
Jan	0.000026	0.4570	0.000088	-0.2	0.667	0.849	0.525
Feb	0.000032	0.3626	0.000089	-0.2	0.660	0.843	0.517
Mar	0.000035	0.2356	0.000073	-0.1	0.654	0.798	0.536
Apr	0.000032	0.2998	0.000079	-0.2	0.649	0.805	0.523
May	0.000035	0.2722	0.000079	-0.2	0.649	0.806	0.522
Jun	0.000034	0.2565	0.000076	-0.2	0.655	0.806	0.532
Jul	0.000031	0.3060	0.000076	-0.2	0.656	0.808	0.532
Aug	0.000028	0.3471	0.000076	-0.2	0.660	0.811	0.536
Sep	0.000028	0.3526	0.000078	-0.2	0.659	0.815	0.533
Oct	0.000027	0.3907	0.000081	-0.2	0.662	0.826	0.530
Nov	0.000023	0.5217	0.000089	-0.2	0.658	0.840	0.515
Dec	0.000033	0.3148	0.000084	-0.2	0.652	0.821	0.518

Statistics for years: 2004 - 2019, linear regression weighted by radio-days squared. 'stdError' is standard error, 'r^2', 'slope' and 'intercept' of the linear regression fit. Survival 'begin' and 'end' are survivals in the first and last year of the period.

Supplementary Note 3. Pinpointing the trend change timing via regression discontinuity analysis

Regression discontinuity analysis was performed to determine whether there was a cutoff point where the trend may have become discontinuous [2], as could occur before and after a specific date or event, with trends having the potential to significantly change in their magnitude and slope. This was performed using the R script *WolfProc.R* as a varying slope regression discontinuity analysis [3–5].

Supplementary Table 9: Regression discontinuity analysis found significant trend cutoff in October, November, and December 2012.

Cut-point	Year				
**Month**	**2010**	**2011**	**2012**	**2013**	**2014**
Jan	0.990 (0%)	0.530 (9%)	0.520 (9%)	0.652 (5%)	0.481 (-7%)
Feb	0.736 (8%)	0.414 (15%)	0.470 (13%)	0.350 (-12%)	0.443 (-10%)
Mar	0.822 (-5%)	0.346 (19%)	0.240 (23%)	0.656 (-7%)	0.731 (-5%)
Apr	0.861 (-4%)	0.646 (8%)	0.223 (21%)	0.736 (-4%)	0.827 (-3%)
May	0.855 (-4%)	0.671 (8%)	0.241 (21%)	0.828 (-3%)	0.955 (-1%)
Jun	0.825 (-5%)	0.728 (6%)	0.205 (22%)	0.751 (-4%)	0.426 (-10%)
Jul	0.774 (-5%)	0.746 (5%)	0.105 (24%)	0.766 (-4%)	0.257 (-13%)
Aug	0.731 (-6%)	0.745 (5%)	0.193 (17%)	0.834 (-2%)	0.284 (-11%)
Sep	0.971 (-1%)	0.637 (7%)	0.095 (21%)	1.000 (0%)	0.523 (-7%)
Oct	0.999 (0%)	0.684 (6%)	0.022* (25%)	0.834 (2%)	0.356 (-9%)
Nov	0.907 (-2%)	0.813 (3%)	0.039* (19%)	0.684 (4%)	0.460 (-6%)
Dec	0.790 (4%)	0.721 (6%)	0.041* (24%)	0.757 (-4%)	0.511 (-7%)

R script tested for the existence of discontinuity cutoffs in each month of five consecutive years. The table cells show the p-value significance of discontinuity, followed in parentheses by the overall wolf mortality percentage change at the discontinuity time. * marks p-values ≤ 0.05.

The R script iterated through each month of five consecutive years and tested for the existence of discontinuity cutoff points, see Supplementary Table 9. The only sharp trend discontinuity cutoff [2] was found spanning October, November, and December 2012, with significant p-values of 0.022, 0.039, and 0.041, respectively, when the mortality trend jumped to a new 22% higher level (an average of the aforementioned 3 months). This timing coincided with the initiation of the first wolf hunting and trapping season of 2012. There are no candidates for any other timing of cutoffs since the p-values became increasingly less significant for the earlier months of 2012, while all columns for years 2010, 2011, 2013, and 2014 have large p-values.

The January row in Supplementary Table 9 shows that statistics aligned with standard calendar years starting on January 1st were particularly unsuitable for noticing sharp discontinuity. Only part of the mortality from the 2012 to 2013 hunting season occurred in November and December 2012, thus producing a smooth transition when counting statistics in calendar years.

Supplementary Fig. 1 graphically illustrates the numerical p-values in Supplementary Table 9. Testing for the cutoff points on November 1st of years 2011 and 2013 showed a low likelihood of such cut point, with p-values of 0.813 and 0.684, respectively, leading to the appearance of Supplementary Fig. 1a and Supplementary Fig. 1c. On the other hand, the p-value was

0.039 for November 2012 and corresponds to a sharp breaking of the trend in Supplementary Fig. 1b.

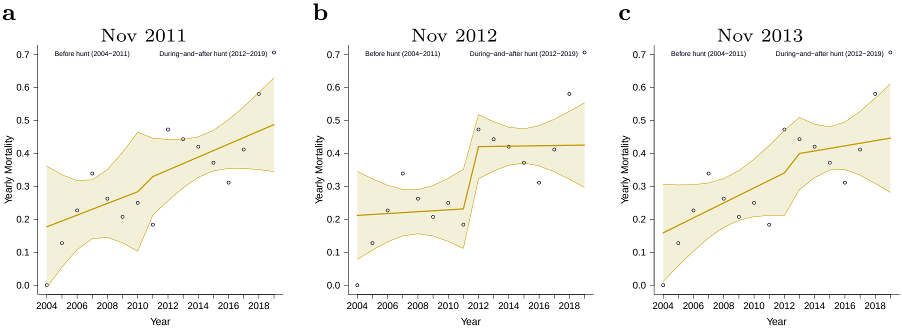

Supplementary Fig. 1: **Graphical illustration of the regression discontinuity test results.**
(a) Trend cutoff November 1st, 2011. **(b)** Trend cutoff November 1st, 2012. **(c)** Trend cutoff November 1st, 2013. With the statistical beginning of the year offset to November 1st, large p-values were produced by discontinuity tests for 2011 and 2013, as reflected in the lack of discontinuity in plots (a) and (c). Plot (b) corresponds to a significantly low p-value (0.039) produced by the test for 2012, the year of hunting initiation (see all values in Supplementary Table 9). The solid beige line shows the mean value of the discontinuous regression, while shaded areas show 95% confidence intervals.

Additional validation scenarios

Thus, November 1st, 2012, being the initiation point for the 2012–2014 wolf hunting seasons, coincides well with the middle of the trend discontinuity cutoff in October–December 2012. A choice of October 2012, with the lowest discontinuity p-value (0.022), would result in a similar trend, with an even higher jump in mortality at the trend cutoff point (see Supplementary Fig. 2).

There is always an uncertainty inherent to radiotelemetry data when accounting for the fate of the censored wolves and the exact dating of the deaths that may lead to mismeasured wolf mortality [6]. Our analysis used the end date in the MN DNR data for the established wolf death date. There may be other choices of adjusting the uncertainty of the death date, as was done by Chakrabarti et al. [7], however we found that for this data set such adjustments have minimal effect on the survival/mortality results, and any adjustment method without the precise timing knowledge remains arbitrary. A validation using the last seen alive date as the mortality date was performed, demonstrating that even with such extreme edge case adjustment the overall mortality would change by only a fraction of a percent (from 34.7% to 35.6%), and confirming a similar trend discontinuity in October–November 2012 (see in Supplementary Table 10). Thus, the end date in MN DNR data set was chosen for consistency of treatment with the censored wolves.

A verification for the trend discontinuity within the during-and-after hunt data intervals is performed in Supplementary Note 4.

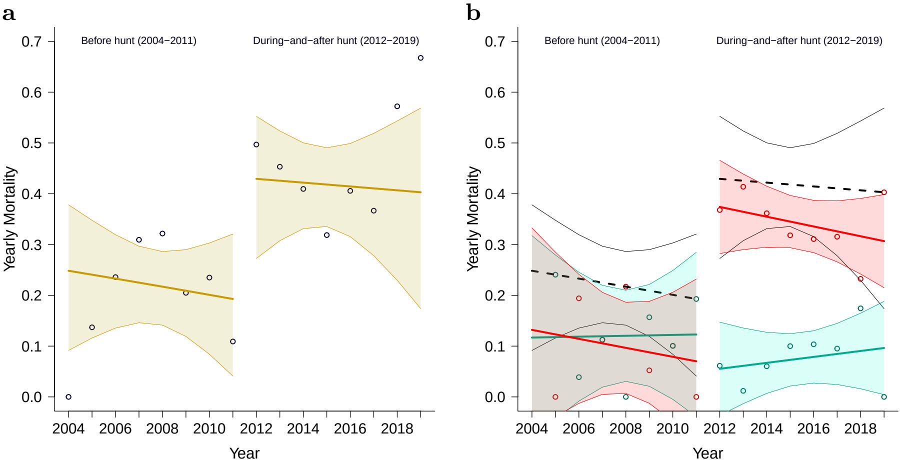

Supplementary Fig. 2: **Wolf mortality for the periods before and after the first hunt, using October 1st, 2012 as the trend cutoff point. (a)** All-cause mortality before and during-and-after hunting seasons. Beige line – trend before and during-and-after hunting seasons indicated near the top of the figure. **(b)** Same as (a), with mortality shared by natural and human causes. Magenta lines and data points – mortality trend by natural causes (red lines) and mortality trend by human causes (data points). Black dashed line – trend and confidence interval outlines the sum of all-cause mortality. All corresponding shaded areas – 95% confidence intervals. For comparison with Fig.2 in the main manuscript.

Supplementary Table 10: Regression discontinuity validation in edge case of using the last seen dates as the dates of death also found significant trend cutoff in October and November 2012.

**Cut-point**	**Year**				
**Month**	**2010**	**2011**	**2012**	**2013**	**2014**
Jan	0.912 (2%)	0.409 (13%)	0.723 (5%)	0.550 (-7%)	0.437 (-9%)
Feb	0.644 (9%)	0.710 (6%)	0.756 (5%)	0.317 (-12%)	0.458 (-8%)
Mar	0.971 (-1%)	0.258 (22%)	0.342 (19%)	0.750 (-5%)	0.841 (-3%)
Apr	0.964 (1%)	0.519 (11%)	0.391 (15%)	0.791 (-3%)	0.903 (-1%)
May	0.971 (1%)	0.520 (11%)	0.393 (15%)	0.880 (-2%)	0.710 (-4%)
Jun	0.931 (1%)	0.357 (11%)	0.165 (17%)	0.999 (0%)	0.523 (-6%)
Jul	0.978 (0%)	0.437 (10%)	0.160 (17%)	0.865 (-2%)	0.232 (-11%)
Aug	0.937 (1%)	0.303 (11%)	0.205 (13%)	0.766 (2%)	0.462 (-5%)
Sep	0.927 (1%)	0.364 (10%)	0.174 (13%)	0.908 (1%)	0.546 (-5%)
Oct	0.623 (6%)	0.284 (11%)	0.046* (17%)	0.833 (2%)	0.230 (-8%)
Nov	0.314 (10%)	0.665 (4%)	0.026* (17%)	0.346 (7%)	0.630 (-3%)
Dec	0.503 (9%)	0.835 (3%)	0.277 (11%)	0.459 (-7%)	0.336 (-8%)

R script tested for the existence of discontinuity cutoff points in each month of five consecutive years. The table cells show the p-value significance of discontinuity, followed in parentheses by the overall wolf mortality percentage change at the discontinuity time. * marks p-values ≤ 0.05.

Supplementary Note 4. Absence of trend discontinuities following termination of the wolf hunting seasons

In Supplementary Note 3 we established the trend discontinuity at the initiation of the first Minnesota wolf hunting season in November 2012. It is interesting to verify whether there is an additional discontinuity after the hunt discontinuation. For this analysis we have only eight yearly data points in the years 2012–2019, and while it is not much data for strong conclusions, we were asked for such analysis as an additional validation. The trend discontinuity analysis was applied to this during-and-after hunt period data (see Supplementary Table 11). To establish a trend, it is required at least three data points, thus allowing to check for regression discontinuity in years 2015 and 2016, with the entire years 2014 and 2017 presented in Supplementary Table 11 only as a reference. There was no indication of significant discontinuity in the years 2015–2016, nor in the year 2014 and most of the year 2017, with expected stochastic shifts due to time of tested discontinuity relative to the reported timing of the wolf deaths over time. There is a significant discontinuity point in November 2017, which indicates a mortality increase by 12%, however there is only two years of sparse mortality data following this time point, such lower confidence denoted by grayed table cells for the beginning of the year 2014 and the end of the year 2017.

Supplementary Table 11: Regression discontinuity test applied to during-and-after hunt data does not indicate trend discontinuity following termination of the hunting seasons.

**Cut-point**	**Year**			
**Month**	**2014**	**2015**	**2016**	**2017**
Jan	0.331 (-37%)	0.728 (-7%)	0.788 (5%)	0.210 (-20%)
Feb	0.752 (16%)	0.763 (-7%)	0.483 (15%)	0.477 (-14%)
Mar	0.805 (15%)	0.245 (-27%)	0.254 (27%)	0.963 (-1%)
Apr	0.774 (15%)	0.264 (-22%)	0.320 (21%)	0.876 (3%)
May	0.735 (18%)	0.159 (-29%)	0.534 (15%)	0.900 (3%)
Jun	0.804 (-11%)	0.304 (-19%)	0.371 (19%)	0.781 (6%)
Jul	0.541 (-18%)	0.527 (-8%)	0.249 (17%)	0.471 (11%)
Aug	0.609 (-14%)	0.537 (-8%)	0.190 (20%)	0.695 (6%)
Sep	0.908 (-3%)	0.194 (-15%)	0.345 (14%)	0.578 (8%)
Oct	0.769 (-6%)	0.497 (-6%)	0.529 (5%)	0.204 (9%)
Nov	0.814 (-4%)	0.541 (-5%)	0.092 (-5%)	0.0023* (12%)
Dec	0.206 (21%)	0.142 (23%)	0.967 (1%)	0.194 (21%)

R script tested for the existence of discontinuity cutoff points in each month of four consecutive years. The table cells show the p-value significance of discontinuity, followed in parentheses by the overall wolf mortality percentage change at the discontinuity time. * marks p-values ≤ 0.05.

Supplementary Note 5. Confirming the consistency of regression when wolf deaths by unknown causes are excluded

Supplementary Table 12 presents the regression analysis performed while discounting 6 out of 59 wolf mortality events due to unknown causes, rather than imputing them as fractional shares of mortalities. Imputing the unknown cause mortality allows us to account for all wolf deaths in the data rather than underestimating wolf mortality, with no noticeable bias in the regression outcome.

Supplementary Table 12: Wolf survival and mortality summary **excluding** the imputation of wolf deaths with unknown causes.

		Yearly mortality				Wolf deaths by cause	
**Period**	**Survival**	**all**	**human**	**natural**	**R-days**	**human**	**natural**
			All wolves				
**Before**	0.801 (0.71-0.90)	19.9%	9.2%	10.7%	21382	6.00	7.00
**After**	0.581 (0.49-0.69)	41.9%	34.5%	7.3%	26946	33.00	7.00
All	0.670 (0.60-0.75)	33.0%	24.3%	8.7%	48328	39.00	14.00
			Adult				
**Before**	0.780 (0.68-0.90)	22.0%	11.0%	11.0%	17614	6.00	6.00
**After**	0.584 (0.48-0.71)	41.6%	32.2%	9.4%	21071	24.00	7.00
All	0.666 (0.59-0.75)	33.4%	23.3%	10.1%	38685	30.00	13.00
			Male				
**Before**	0.754 (0.61-0.93)	24.6%	14.1%	10.6%	9035	4.00	3.00
**After**	0.590 (0.45-0.78)	41.0%	23.4%	17.6%	9706	8.00	6.00
All	0.664 (0.56-0.79)	33.6%	19.2%	14.4%	18741	12.00	9.00
			Female				
**Before**	0.808 (0.67-0.97)	19.2%	7.7%	11.5%	8579	2.00	3.00
**After**	0.579 (0.45-0.75)	42.1%	39.6%	2.5%	11365	16.00	1.00
All	0.668 (0.56-0.79)	33.2%	27.1%	6.0%	19944	18.00	4.00
			Juvenile				
**Before**	0.908 (0.75-1.10)	9.2%	0.0%	9.2%	3768	0.00	1.00
**After**	0.571 (0.40-0.82)	42.9%	42.9%	0.0%	5875	9.00	0.00
All	0.685 (0.54-0.87)	31.5%	28.4%	3.2%	9643	9.00	1.00

Wolf hunting seasons always commenced in November of wolf hunting seasons 2012–2014; therefore, all statistics are offset to start each year on November 1st. Periods before hunting include 2004 to October 31, 2012, while periods during-and-after hunting commenced include November 1st, 2012 to the end of 2019. All years were calculated for the entire data period, combining before and during-and-after periods. The range in braces following 'Survival' is a 95% confidence interval range. R-days stands for the number of radiotelemetry days for each time interval. Fractional wolf death counts are the result of six wolf deaths with unknown causes, imputed proportionately to the known mortality causes for each period.

See the comparisons between Supplementary Table 12 and corresponding Table 1 (where imputation was performed), which show that the patterns appear qualitatively the same between the two representations, with 10% lower values across all numbers where the unknown deaths were omitted, thereby showing the benefit of this imputation.

Supplementary References

[1] Sheather, S.J.: Weighted Least Squares, pp. 115–123. Springer, New York, NY (2009). https://doi.org/10.1007/978-0-387-09608-7_4

[2] Jacob, R., Zhu, P., Somers, M.-A., Bloom, H.: A practical guide to regression discontinuity. MDRC (2012)

[3] Hanck, C., Arnold, M., Gerber, A., Schmelzer, M.: Introduction to econometrics with R. University of Duisburg-Essen, 1–9 (2019)

[4] R Companion to Real Econometrics (accessed December 14, 2022). https://bookdown.org/carillitony/bailey/ (2021)

[5] Bailey, M.A.: Real Econometrics: The Right Tools to Answer Important Questions. Oxford University Press, Oxford, U.K. (2019)

[6] Treves, A., Artelle, K.A., Darimont, C.T., Parsons, D.R.: Mismeasured mortality: correcting estimates of wolf poaching in the United States. Journal of Mammalogy **98**(5), 1256–1264 (2017). https://doi.org/10.1093/jmammal/gyx052

[7] Chakrabarti, S., O'Neil, S.T., Erb, J., Humpal, C., Bump, J.K.: Recent trends in survival and mortality of wolves in Minnesota, United States. Frontiers in Ecology and Evolution **10** (2022). https://doi.org/10.3389/fevo.2022.826358
